# The Diet Quality of Ultramarathon Runners Taking Part in an Australian Event: A Cross-Sectional Explorative Study

**DOI:** 10.3390/nu17030485

**Published:** 2025-01-29

**Authors:** Joel C. Craddock, Gabriel Walker, Michael Chapman, Kelly Lambert, Gregory E. Peoples

**Affiliations:** 1 School of Medical, Indigenous and Health Sciences, Faculty of Science Medicine and Health, University of Wollongong, Wollongong, NSW 2500, Australiaklambert@uow.edu.au (K.L.); 2 My Health Pledge, Kittani St, Kirrawee, NSW 2232, Australia; 3 Graduate School of Medicine, Faculty of Science Medicine and Health, University of Wollongong, Wollongong, NSW 2500, Australia; peoples@uow.edu.au

**Keywords:** runner, ultrarunner, diet quality, diet quality index, DQI, dietary pattern, nutrients, exercise, performance

## Abstract

**Background/Objectives**: Ultramarathon runners exceed the physical activity guidelines and in doing so are constantly exposed to physical and metabolic demands, requiring strategic dietary practices to support training, performance, and recovery. This study aimed to assess the diet quality and nutrient intake in runners enrolled in an Australian-based ultramarathon. **Methods**: A 3-day food diary was collected using the Australian smartphone application ‘Easy Diet Diary’ during both peak and taper periods. Macronutrient and micronutrient intakes were analysed using the AUSNUT 2011–2013 food composition database within the Foodworks professional software, and diet quality was evaluated using the Healthy Eating Index for Australians (HEIFA-2013). **Results**: A total of 26 runners participated in the study. The results revealed that, although runners met or exceeded protein recommendations, their carbohydrate intake fell short of endurance-specific guidelines, whilst total dietary fat intake exceeded recommendations (excluding long-chain polyunsaturated fatty acids). Diet quality scores averaged 63.1 out of 100, reflecting moderate alignment with dietary recommendations. Fruit, vegetable, and wholegrain food groups were inadequately consumed. **Conclusions**: The findings indicate that ultramarathon runners who easily exceed physical activity recommendations, may paradoxically consume suboptimal diets, characterized by insufficient intake of core food groups such as whole grains and fruits, alongside excessive consumption of discretionary items. This dietary pattern may not only elevate their risk of chronic disease but also impair optimal performance by compromising recovery and adaptation to training. Further research is warranted to better understand the dietary behaviors and nutritional needs of this population.

## 1. Introduction

The popularity of ultra-marathon running has surged considerably in the last two decades [1]. Ultra-distance running, defined as any race exceeding the marathon distance of 42.195 km or lasting more than six hours to completion, requires prolonged and intense exertion, exposing athletes to significant physiological, mechanical, and metabolic stresses [2,3]. Both the training for and participation in these events substantially exceeds the world health physical activity recommendations [4], and in doing so demands substantial energy intake to support performance and recovery [5]. During ultra-endurance events, energy expenditure is recommended to range from 150 to 400 kcal per hour, which can result in a total daily requirement of several thousand calories depending on the event duration [6]. Meeting these elevated energy needs is essential not only for maintaining running performance but also for overall health and recovery. Failing to do so can result in muscle mass loss, weakened immune function, and diminished endurance capacity [7].

Carbohydrates (CHO) are particularly important in endurance sports due to their role in energy metabolism. Muscle and liver glycogen are key fuel sources, and maintaining adequate glycogen levels before, during, and after exercise helps to sustain performance and promote recovery [8]. Research consistently shows that sufficient CHO intake allows endurance runners to prolong their running capacity by sustaining energy levels [9,10,11] and may assist with exercise induced muscle damage [12]. Current recommendations suggest that ultramarathon runners should aim for 7–12 g/kg/day of CHO during training, and up to 120 g/h during events to sustain energy needs [13,14]. However, many athletes restrict wholefood CHO sources both before and during races and training sessions due to their high fibre content [13] which can contribute to gastrointestinal discomfort. Some literature suggests that up to 82% of ultrarunners experience this issue [15].

Protein consumption has historically received less attention among endurance athletes compared to carbohydrates, despite its importance. Protein intake in endurance athletes supports muscle repair, minimises muscle protein breakdown, contributes to a positive nitrogen balance, and improves glycogen replenishment all of which can enhance overall running performance during prolonged activity [16,17]. To optimise performance, endurance athletes are recommended to consume between 1.2 to 2 g of protein per kilogram of body weight per day, ideally in intervals of 3 to 4 h [18].

To meet their high energy and macronutrient demands, including carbohydrate and protein needs while minimising fibre intake, many ultramarathon athletes rely heavily on sports-specific nutrition products such as drinks, gels, and bars. A study conducted by Heikura et al. (2018) reported that of 104 elite male and female middle- and long-distance competitive runners, 58% consumed carbohydrates during training with 90% of these athletes relying solely on sports nutrition products. These specialised products were in the form of drinks, gels, chews, and bars to meet their carbohydrate intake needs during training and events [19]. Whilst this approach to fuelling may be convenient and ideal for performance, consistent reliance on highly processed sports nutrition products could reduce the variety of foods in athletes’ diets, potentially impacting overall diet quality.

Diet quality refers to the extent to which an individual’s dietary intake aligns with established nutritional guidelines aimed at promoting health and preventing disease. It underscores the importance of consuming a diverse range of nutrient-dense foods while minimising the intake of processed and low-nutrient options [20]. Commonly used measures to evaluate diet quality include indices such as the Healthy Eating Index (HEI) and the Mediterranean Diet Score, both of which assess adherence to dietary recommendations [21,22]. The Healthy Eating Index for Australian adults (HEIFA-2013) is another validated index which aligns with the 2013 Australian Dietary Guidelines assessing both food and nutrient intake. The HEIFA-2013 provides a score between 0 and 100, reflecting adherence to these guidelines. Scores are based on 19 components, including core foods (fruits, vegetables, grains, meats, dairy) and negative nutrients considered harmful to health, such as saturated fat, added sugars, sodium, and alcohol [23]. Higher scores are associated with greater intake of core foods and healthy fats and lower intake of discretionary foods (Table 1). Unlike some other diet quality indices, such as the Australian Food Reference Score [24], which is scored out of 73 and categorises scores into qualitative ranges (e.g., <33 as “Needs work”, 39–46 Excellent), the HEIFA-2013 does not assign qualitative categories, instead providing a total score out of 100 to indicate adherence to dietary guidelines. Notably, achieving a perfect score of 100 is not imperative; rather, higher scores indicate better adherence to dietary guidelines through greater consumption of core foods and healthy fats alongside reduced intake of discretionary foods.

High diet quality that is aligned with healthy eating principles has been consistently associated with reduced all-cause mortality and a lower risk of developing non-communicable diseases, including cardiovascular disease (CVD), type 2 diabetes (T2D), and cancer [25,26,27]. Surprisingly, endurance athletes may exhibit more advanced atherosclerosis and greater myocardial damage than sedentary individuals [28,29,30]. If ultramarathon runners are relying heavily on sports nutrition products for prolonged periods, which research suggests may be the case [19], they may be inadvertently impacting their health in a negative way. As underscored within a recent scoping review conducted by our team [31], the research examining diet quality in ultramarathon runners using validated diet quality indices is scarce. Given the importance of diet for both performance and health, this study aimed to assess the diet quality of ultramarathon runners, who were taking part in an Australian event, during peak and taper training periods. A secondary objective was to evaluate whether performance-focused nutrition strategies align with the recommended guidelines for ultra-endurance athletes.

## 2. Materials and Methods

### 2.1. Study Design and Overview

This cross-sectional study of diet quality was a secondary component of an online survey evaluating the nutrition knowledge of participants competing in an ultra-endurance running event held in Australia in May 2023. Invitations to complete the online nutrition knowledge survey were sent to all registered participants in the ultra-running event. The final question invited those interested to participate in the current study focussing on diet quality. Participants who provided their email addresses constituted the recruitment pool for this current research study.

Dietary intake was collected on two occasions. The first occasion was three–four weeks prior to the ultra-endurance event to capture the ‘peak training’ period, and the second occasion three days prior to the event to capture the ‘taper’ period. The study was conducted in line with the Code of Ethics of the World Medical Association [32]. Ethics were approved by the University of Wollongong Human Research Ethics Committee (2022/346). As this was an exploratory study, a power calculation was not performed to determine the required sample size. Participants provided tacit consent through the disclosure of their email addresses to receive study information and participate and were free to retract their participation and data at any time.

### 2.2. Participants and Demographics

Participants were recruited via online survey. To establish a healthy comparator group, runners who participated in the 11 km and 22 km race distances were included, as they did not meet the criteria for ultra-distance running (events exceeding 42 km). These runners were combined to form Group A (healthy comparator group), while competitors in the 50 km and 100 km distances were assigned to Groups B and C, respectively. Eligibility for the study extended to competitors from all four race distances (11 km, 22 km, 50 km, and 100 km) provided they were 18 years of age or older and had officially registered for the event. Both male and female competitors were eligible for inclusion. Demographic data including age, gender, training history, level of education, nutrition training/education, household income, and distance they planned to complete were obtained from the nutrition knowledge survey.

### 2.3. Dietary Data Collection

Dietary data were collected using the validated Australian smartphone application “Easy Diet Diary” [33]. The research team, which included an accredited practising dietitian (APD), provided information on how to download the application and log their dietary intake. Information including the level of detail required was included, as well as examples depicting how a meal would be logged being provided. Participants were instructed to record three days of intake (two weekdays and one weekend day) during two separate phases of their training for the event (peak and taper).

### 2.4. Data Analysis

Data were analysed using the AUSNUT 2011–2013 food composition database within the Foodworks professional software (version 10, Xyris Software, 2012). The “Easy Diet Diary” application uses a combination of both the AusFoods database, which derives its data from AUSNUT 2011–2013, and the AusBrands database, which derives its data from food labels [34]. Consequently, foods and beverages from the AusBrands database do not have complete nutrient profiles. In such cases, items with incomplete profiles were substituted with the best fit option from the AUSNUT 2011–2013 database by researcher GW through food and nutrient comparisons. A secondary member of the research team (an accredited practising dietitian) reviewed a 20% random sample for quality assurance. The AUSNUT 2011–2013 dietary supplement nutrient database was used to determine nutritional composition of reported supplement intake [35].

### 2.5. Diet Quality

The “Healthy Eating Index for Australian adults” (HEIFA-2013) was used to assess the diet quality of participants [23]. Due to the food group categorisation level available within the Australian Dietary Guidelines database [36], a modified version of the HEIFA-2013 was implemented. In this modified version, the categories for vegetable variety scoring that originally were cruciferous, tuber or bulb were changed to starchy, whole, and brassica, each still contributing up to one point towards the total score. To quantify intake, a matrix was developed in Microsoft Excel (version 16.76) using the Australian Dietary Guidelines database, which provides serving sizes per 100 g for core food groups across 5740 items in the AUSNUT 2011–2013 database. This detailed categorisation allowed for aggregation based on HEIFA-2013 scoring criteria; for instance, total fruit servings were calculated by summing dried and fresh/canned fruit values [37]. Discretionary food items, not included in the existing database, were manually identified, and their serves were calculated by dividing the kilojoules per 100 g by 600 kJ to determine serves aligning with previous research [38]. Additional calculations for negative nutrient intake were performed in Microsoft Excel. The percentage of total energy from saturated fat was determined by multiplying daily saturated fat intake in grams by 37.7 kJ, dividing this by total daily energy intake, and multiplying by 100. Finally, each HEIFA-2013 domain was totalled for each participant and divided by the number of recall days to provide daily intake values. Table 1 provides an overview of the scoring matrix.

### 2.6. Statistical Analysis

Prior to analysis, data cleaning was performed in Microsoft Excel (Version 16). Where participants average daily energy intake was less than 4000 kJ or exceeding 40,000 kJ, these data were omitted for analysis aligning with similar research and physiological plausibility [39]. Data were analysed using Prism GraphPad software (Version 10) [40]. The Shapiro–Wilk test assessed normality. One-way ANOVA testing was used for parametric samples, whilst Kruskal–Wallis testing was used for non-parametric samples between groups. Unpaired *t*-tests were applied to determine which groups were significantly different. Parametric data were reported as means with 95% confidence intervals, while non-parametric data were reported as medians with 25th to 75th quartiles.

## 3. Results

### 3.1. Participants

**Figure 1:**
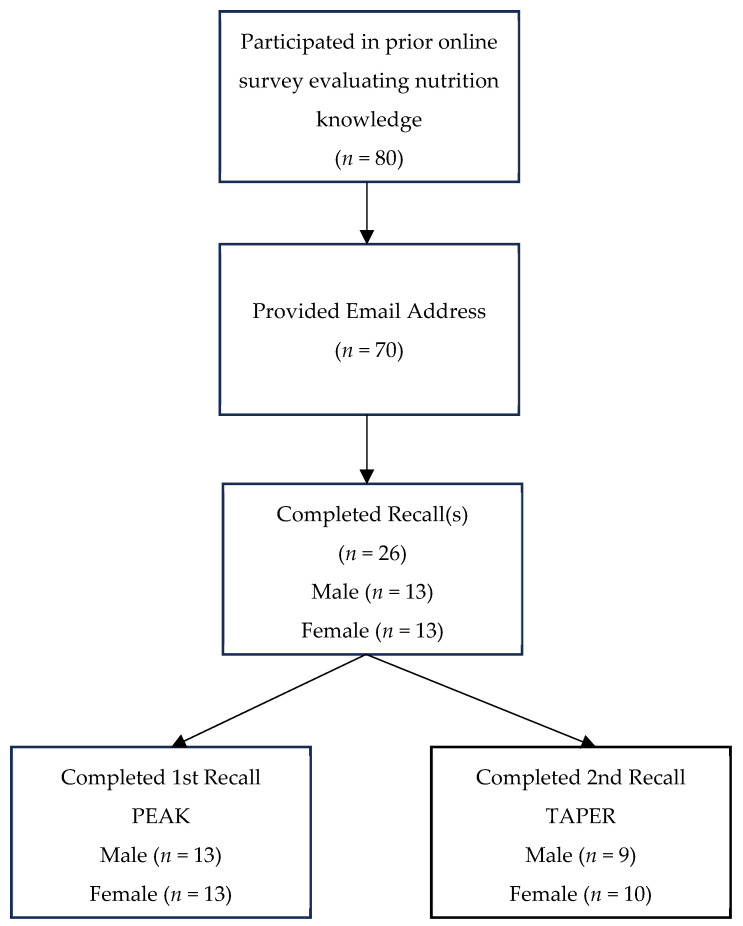
Flow chart of participants.

Of the 70 runners who expressed interest in the study by providing their email addresses, 26 completed dietary recalls (37%), comprising 13 males and 13 females (Figure 1). Among these participants, three competed in the 11 km or 22 km events, 15 in the 50 km event, and eight in the 100 km event. During the peak training period, 26 participants provided dietary data, averaging 2.24 days of recall, while 19 participants provided dietary data during the taper period, averaging 1.5 days of recall. All participants average daily energy intake was between 4000 kJ and 40,000 kJ and was therefore included in the analysis. An overview of participants demographics is provided in Table 2.

### 3.2. Diet Quality

During the peak training period, median HEIFA-2013 scores (out of 100) were as follows: group A: 59.6 (47.2–72.0 IQR), group B: 57.5 (48.6–66.4 IQR), and group C: 59.9 (54.0–65.8 IQR, Table 3). In the taper period, scores changed to 56.2 (46.14–66.2 IQR) for group A, 55.4 (48.3–62.6 IQR) for group B, and 61.4 (52.3–70.4) for group C. Most domains of diet quality within the HEIFA-2013 were comparable within groups between peak and taper periods. Athletes in groups B and C exhibited low scores in the domains of whole grains, total fruits, and fruit and vegetable variety. They also scored poorly in discretionary foods, sodium, and saturated fat, indicating excessive intake. Conversely, the cohort achieved high scores for total cereals, total vegetables, meat and alternatives, dairy and alternatives, as well as monounsaturated and polyunsaturated fatty acids. Additionally, all groups had low intakes of added sugars and alcohol.

### 3.3. Estimated Macronutrient Intake

During the peak and taper training phases median (IQR) daily energy intake was, respectively, 8477 kJ (Table 4, 4747–13,497 kJ) and 6371 kJ (5300–6504 kJ) for group A; 10,233 kJ (7365–11,423 kJ) and 12,333 kJ (8676–14,784 kJ) for group B; and 11,628 kJ (6864–15,198 kJ) and 9809 kJ (8562–12,330 kJ) for group C. Median (IQR) daily carbohydrate intake in grams per kilogram of body weight was low across all groups with 2.2 g/kg (Table 4, 0.8–3.5 g/kg) and 1.7 g/kg (1.4–2.3 g/kg) for group A; 4.0 g/kg (2.7–5.4 g/kg) and 4.5 g/kg (2.9–6.2 g/kg) for group B; and 4.0 g/kg (2.4–5.6 g/kg) and 3.9 g/kg (2.7–5.2 g/kg) for group C, respectively, for peak and taper periods. Daily protein intake in grams/kg was 1.3 (0.5–2.2) and 1.1 (0.2–2.0) for group A; 1.6 (1.2–2.5) and 1.9 (1.4–2.5) for group B; and 1.9 (1.3–2.5) and 2.1 (1.6–2.6) for group C across peak and taper training periods, respectively. Total fat intake as a % of total energy was 43% (33–54) and 42% (37–47) in group A, 34% (30–38) and 36% (32–40) in group B, and 37% (30–45) and 35% (24–45) in group C during peak and taper training periods (Table 4).

During the peak training period, no significant differences were observed for energy and macronutrient intake levels between groups (Table 4). However, cholesterol intake was significantly lower in group B compared to groups A and C. During the taper training period, groups B and C exhibited significantly higher consumption of energy, carbohydrates, magnesium, and iron relative to group A. Additionally, group C derived a greater proportion of energy from protein than group B. Group B also demonstrated significantly higher energy intake per kilogram of body weight, as well as increased fat and carbohydrate consumption per kilogram of body weight in comparison to group A (Table 4). Low intake of long chain polyunsaturated fatty acids (docosahexaenoic acid; DHA, and eicosapentaenoic acid; EPA) were also observed with each time period across all groups. Within each group, no significant differences were observed in energy intake, energy intake per kilogram of body weight, or macronutrient intake between the peak and taper training periods.

## 4. Discussion

To our knowledge this is the first study to evaluate the diet quality of ultramarathon runners competing in an ultra-endurance running event held in Australia using a validated diet quality index. We hypothesised that the diet quality of ultramarathon runners would be suboptimal, assuming that prioritisation of ergogenic macronutrient intake would detract from overall diet quality. In this line of thought we also hypothesised that ultramarathon runners would have a high level of compliance with performance-centric macronutrient intake recommendations. Our hypothesis was partially supported as we found our cohort to have poor compliance with the Australian Dietary Guidelines and strong compliance with protein intake recommendations. However, contrary to our hypothesis, the cohort achieved poor compliance with total daily carbohydrate recommendations and exceeded those for total dietary fats (excluding long-chain polyunsaturated fatty acids).

In this study, the highest and lowest HEIFA-2013 scores were observed during the taper phase in which the highest (median 61.4 (52.3–70.4 IQR) was seen in group C (100 km) and the lowest (median 55.43 (48.3–62.6 IQR) was observed by group B (50 km). There is a global scarcity of literature employing diet quality indices (DQIs) in athlete diet assessment and the use of HEIFA-2013 is no exception. However, the HEIFA-2013 was utilised previously in studies with general Australian adult populations. Grech et al. (2017) reported mean scores of 44.6 and 46 for generally healthy Australian men and women aged 35–44 and 45–54, respectively [41]. The finding that our cohort of endurance athletes achieved higher DQI scores than individuals of a similar age group within the Australian general public is supported by existing literature.

A 2020 study by Capling et al. found that their cohort of elite Australian athletes to have higher diet quality than that of the Australian public as assessed by the Athlete Diet Index [42]. The disparity in diet quality between our cohort and the general public mirrored that of Capling et al. and may be attributed to the emphasis that athletes place on dietary optimisation for performance gains, coupled with the increased likelihood that athletes will seek professional dietary guidance, resulting in higher nutrition literacy [42]. In the case of the study of Capling et al., their cohort consisted of athletes from Australian state-based sporting organisations who likely had access to sports dietitians potentially explaining the higher diet quality when compared to general population groups. Although diet quality scores in our cohort were higher compared to studies of the Australian public, this does not diminish concerns about the suboptimal scores in this study which may be associated with poorer long term health outcomes. The subpar diet quality scores observed among ultramarathon runners in this study align with the findings of our team’s previous scoping review, which highlighted generally suboptimal diet quality among athletes within a range of sporting modalities [31].

Grech et al. (2017) identified significant dietary inadequacies within the Australian general public, with their cohort scoring lowest in the HEIFA-2013 domains for total grains, wholegrains, fruits and vegetables (including variety), and fats, indicating higher intake of saturated fats and inadequate intake of monounsaturated and polyunsaturated fatty acids. Highest scores were achieved for total vegetable, meat and alternatives, dairy and alternatives, added sugar, and alcohol intake [41]. Apart from lower intake of total grains and monounsaturated and polyunsaturated fats, these patterns mirror those exhibited by participants in this study. Similarly, patterns of dietary inadequacy in this study also align closely with those of international athletes. A 2022 study by Gacek et al. found their cohort of elite Polish team-sport athletes consumed low quantities of fruits, vegetables, and wholegrains [43]. These findings suggest that ultramarathon runners taking part in this Australian event share similar dietary shortcomings with both the general public and international athletes.

It is important to note that inadequate intake in the athletes of the present study may carry significant negative health implications. Insufficient consumption of fruits and wholegrains has been linked to the development of chronic diseases, including CVD, cancer, T2D, and hypertension [44,45]. Additionally, high intake of sodium, saturated fat and discretionary food items are correlated with higher incidence of obesity, T2D, CVD, dementia, and cancer [46,47,48]. Paradoxically, despite exceeding the WHO guidelines for physical activity [4] by a substantial margin, these athletes are not meeting dietary recommendations essential for long-term health, highlighting the potential risks posed by these inadequacies.

An individualized approach to nutrition is optimal for ultra-endurance runners, as each athlete’s needs vary based on factors such as training load, body composition, and personal preferences. However, from our findings, marathon runners commonly under-consume core food groups, particularly wholegrains and fruits, while eating a limited variety of vegetables and fruits. Additionally, their intake of discretionary items high in sodium and saturated fats is excessive. To optimize performance and recovery, athletes should focus on increasing their consumption of wholegrains, fruits, and vegetables, while reducing reliance on high-sodium and high-fat discretionary foods.

According to the International Society of Sports Nutrition Position Stand: Nutritional Considerations for Single-Stage Ultra-Marathon Training and Racing, approximately 60% of total energy for frequent endurance training should be derived from carbohydrates, 25% from fat, and 15% from protein. [6]. This statement underscores the importance of a high carbohydrate intake in endurance sports given the integral role that carbohydrates play in performance and recovery [18]. In our study, group B (50 km) achieved median carbohydrate intakes of 4.04 and 4.52 g/kg/day during the peak and taper phases, respectively, whilst group C (100 km) had intakes of 3.95 and 3.85 g/kg/day. This shortfall aligns with existing literature, suggesting that ultramarathon runners should aim for 7–12 g/kg/day of CHO during training [13,14]. Wardenaar et al. (2015) investigated male and female competitors (average age 46.5 ± 7.2) training between one and two hours per day during their preparation for either a 60 or 100 km ultramarathon. The study found male competitors to consume an average carbohydrate intake of 4.4 g ± 1.3 kg/day, whilst female competitors consumed on average 4.5 g ± 1.3 kg/day. Both fell short of the suggested 5–10 g/kg daily intake for those training one–two hours per day [49]. Additionally, Baranauskas et al. (2015) concluded that 80.8% of their cohort of endurance athletes failed to meet carbohydrate requirements appropriate to their level of activity [50].

Athletes typically underreport their intake by 10–20%, potentially explaining the lower carbohydrate intakes observed in both the current and previous studies [51,52]. Some endurance athletes intentionally limit carbohydrate intake during training, as this practice may enhance metabolic adaptations in skeletal muscle and shift substrate utilisation towards fat—an approach known as “train low, compete high”, which could also increase glycogen storage capacity [53]. This strategy may help elucidate the frequent suboptimal carbohydrate intake among endurance athletes, which could negatively impact overall diet quality; however, further research is needed. Additionally, the use of diet diaries in our cohort may contribute to underreporting due to social desirability bias [54] or impracticality during training periods. With an average recall of 2.24 days during peak training and 1.5 days during tapering, which is below the recommended minimum of three days, accuracy may be compromised [54]. A lack of understanding by the participants of the nutritional needs for ultramarathon running could also play a role in the low carbohydrate intake observed [6]. Inadequate carbohydrate intake can lead to glycogen depletion, hindering adaptation to physical loads and impairing performance [50]. Furthermore, exercising in a glycogen-depleted state can reduce immune function, increasing the risk of illness and overtraining [6]. To optimise performance and mitigate these risks, endurance athletes should prioritise adequate carbohydrate intake, particularly during intensive training phases. Additionally, implementing structured nutritional education could enhance athletes’ understanding of their nutritional needs, ultimately improving their diet quality and performance outcomes.

The ISSN position statement on nutritional considerations for single-stage ultra-marathon training and racing recommends a protein intake between 1.3 and 2.1 g/kg/day for endurance athletes [6]. Similarly, Vitale et al. (2019) recommend intake of between 1.2–2 g/kg/day [6,18]. In our cohort during the peak training phase, group B (50 km) achieved a median protein intake of 1.56 g/kg/day during the peak training phase and 1.91/kg/day during the taper. Group C (100 km) consumed 1.87 and 2.07 g/kg/day during peak and taper, respectively. These intakes align comfortably with recommendations. Similarly Baranauskas et al. (2015) found elite Lithuanian endurance runners had an average protein intake of 1.6 g/kg/day during the training phase prior to competition [50].

The prioritisation of protein adequacy by athletes may be due to the commonly held belief that protein is advantageous for athletic pursuits [55]. Protein plays an important role in recovery, aiding in repairing muscle damage sustained during exertion and in turn helping to attenuate injury risk [6]. Despite our cohort achieving adequate daily protein intake levels with similar findings reported in other studies involving endurance athletes [50,56], it remains essential to encourage endurance athletes to regularly monitor their protein intake. This is particularly important during intensive training and tapering phases, to ensure they stay within recommended ranges. Of note, protein adequacy may not be achieved in all subgroups of endurance athletes. Female endurance athletes [57], along with individuals following certain dietary patterns such as plant-based diets [58], may be particularly susceptible to inadequate protein intake, although this was not observed in the current study.

Vitale et al. (2019) recommend that endurance athletes derive no less than 20% of their total energy from fats to minimise the risk of deficiency of fat-soluble vitamins, carotenoids, and essential fatty acids [18]. The ISSN suggests a 25% contribution of energy from fat to be optimal for ultramarathon performance [6]. In our study, participants in group B (50 km) derived 34% (95% CI: 30.0–37.9) and 36% (95% CI: 31.7–39.6) of their energy from fat during the peak and taper training phases, respectively, whilst group C (100 km) derived 37% (95% CI: 29.8–45.1) and 34% (95% CI: 24.3–44.7) during the same phases. These intakes exceed recommendations. This parallels the findings by Baranauskas et al. (2015) who assessed that their ultramarathon runners derived 43.1% of their energy from fat during the build-up to events [50]. Our cohort consumed high quantities of monounsaturated, polyunsaturated, and saturated fat. Whilst the elevated intake of poly- and monounsaturated fat is likely favourable, the high saturated fat intake is of concern as this has been shown to increase the risk of dyslipidaemia, hypertension, and CVD [48].

Moreover, the self-reported intake of EPA + DHA in this cohort would likely classify them as having a low overall omega-3 status, consistent with previous omega-3 descriptions of Australian endurance triathletes, whether following an omnivorous or vegan diet [59]. Current recommendations from peak bodies such as the Academy of Nutrition and Dietetics suggest a daily intake of 500 mg of EPA + DHA for optimal health [60]. In contrast, the IOC recommends a higher intake of 2000 mg of omega-3 fatty acids per day for athletes, though this may include ALA alongside EPA and DHA [61]. Addressing both saturated fat and omega-3 intake may be crucial for improving cardiovascular health and overall durability in ultramarathon runners.

This study offers novel insights into the diet quality of ultramarathon runners. Using the validated HEIFA-2013 DQI, based on the latest Australian Dietary Guidelines, allows for evidence-based evaluation of intake that considers gender differences. Unlike some nutrient-based DQIs, HEIFA-2013 is food-based, providing a comprehensive view of diet quality by emphasising whole foods and variety [23]. The “Easy Diet Diary” application enhances data collection by offering detailed logging options for each food item, including type, cooking method, and serving size. This specificity improves the accuracy of nutritional data and the reliability of findings. Moreover, real-time logging reduces memory-related errors common in 24 h recalls and food-frequency questionnaires.

While the study presents valuable insights, there are some limitations to note. A minimum of three days of diet recall is recommended to accurately capture usual intake [54], which was not achieved in our study. Additionally, the participant number was modest, which may influence the generalisability of our findings. Subjective decisions regarding food entry into the Easy Diet Diary application may have misrepresented true food intake, potentially affecting intake representation. Lastly, the HEIFA-2013 awards points based on the percentage of total energy from saturated fats and added sugars rather than total intake, which could obscure unhealthy consumption levels given the elevated energy requirements for endurance-based athletes.

## 5. Conclusions

The findings of this study suggest that ultramarathon runners may consume suboptimal diets that are not aligned with the Australian Dietary Guidelines. These runners under-consumed several core food groups, including wholegrains and fruits, ate a limited variety of fruit and vegetables, and had excessive intake of discretionary items high in sodium and saturated fats. These are areas to be addressed within this cohort to promote health and wellbeing and reduce the risk of future chronic disease. In relation to macronutrient intake, participants in this study failed to meet performance-centric guidelines for carbohydrates but succeeded in meeting protein recommendations. This highlights a possible knowledge deficit pertaining to the nutritional requirements of the sport, indicating that nutritional education is likely to be of use.

## Figures and Tables

**Table 1:** Modified HEIFA-2013 scoring matrix.

Component and Points Available	Criteria for Maximum Score	Criteria for Minimum Score
Discretionary foods—10	Male: ≤3 serves Female: ≤2.5 serves	Male: >6 serves Female: >5.5 serves
Total vegetables—5	Male: ≥6 serves Female: ≥5 serves	Both genders: <1 serve
Green and brassica vegetables—1	≥1 serve	<1 serve
Orange vegetables—1	≥1 serve	<1 serve
Starchy vegetables—1	≥1 serve	<1 serve
Whole vegetables—1	≥1 serve	<1 serve
Legumes—1	≥0.5 serve	<0.5 serve
Total fruit—5	≥2 serves	<0.5 serve
Fruit variety—5	2 or more varieties per day	<2 varieties per day
Total cereals—5	≥6 serves	<1 serve
Wholegrains—5	≥3 serves	<1 serve
Meats and alternatives—10	Male: ≥3 serves Female: ≥2.5 serves	Male: <1 serve Female: <0.5 serve
Dairy and alternatives—10	≥2.5 serves	<0.5 serve
Water—5	≥50% of total beverage volume	<10% of total beverage volume
Saturated fat—5	≤10% of total energy	>12% total energy
Combined MUFA & PUFA—5	Male: ≥4 serves Female: ≥2 serves	Male: <1 serve Female: <0.5 serve
Sodium—10	<1610 mg	≥2300 mg
Added sugars—10	<15% of total energy	>20% of total energy
Alcohol—5	≤2 standard drinks per day	>2 standard drinks per day

If not specified, scoring is applicable to both genders. MUFA: monounsaturated fatty acid, PUFA: polyunsaturated fatty acid.

**Table 2:** Anthropometric characteristics of participants (*n* = 26).

	Group A (11 and 22 km) *n* = 3	Group B (50 km) *n* = 15	Group C (100 km) *n* = 8
Age (years)	43 (31.1–54.8)	43.6 (37.1–50.1)	43.3 (34.6–51.9)
Body mass (kg) ^a^	83.2 (58.6–97.8)	70 (57.9–80.3)	66.5 (62–78.5)
Height (m)	1.7 (1.4–2.0)	1.74 (1.7–1.8)	1.75 (1.7–1.8)
BMI (kg·m^2^) ^a^	26.0 (22.4–32.3)	22.73 (21.1–25.2)	22.11 (21.7–23.1)
Highest level of Education (*n*)			
Certificate/diploma	-	-	3
Bachelor’s degree	2	10	4
Postgraduate degree	1	5	1
Weekly Training Km (*n*) ^b^			
0–19	-	-	-
20–39	1	4	-
40–59	-	4	6
60–79	1	4	1
80–99	1	3	-

^a^ Non-parametric data expressed as median (25–75 percentile). ^b^ One participant did not report weekly training kilometers.

**Table 3:** Diet quality measured by the Healthy Eating Index for Australian Adults (HEIFA-2013) for endurance athletes in groups A, B, and C during the peak and taper training periods.

	HEIFA Points Available	Criteria for Maximum Score	Criteria for Minimum Score	Group A (11 and 22 km) PEAK	Group A (11 and 22 km) TAPER	Group B (50 km) PEAK	Group B (50 km) TAPER	Group C (100 km) PEAK	Group C (100 km) TAPER
Discretionary	10	Male: ≤3 serves Female: ≤2.5 serves	Male: >6 serves Female: >5.5 serves	8.8 (5.6–10)	10.0(5.0–10.0)	2.5 (0.00–10)	1.2 (0.0–5.0)	5 (2.5–8.8)	5.00 (2.5–7.5)
Total vegetables	5	Male: ≥6 serves Female: ≥5 serves	Both genders: <1 serve	3 (2–4.8)	3.0 (3.0–4.0)	4 (2.5–5)	5.0 (2.3–5.0)	3.5 (2.3–5)	3.0 (1.5–4.5)
Green and brassica vegetables	1	≥1 serve	<1 serve	0.5 (0.0–1.0)	0.0 (0.0–1.0)	0.0 (0.0–1.0)	0.0 (0.0–0.0)	0.00 (0.00–0.8)	0.0 (0.0–0.5)
Orange vegetables	1	≥1 serve	<1 serve	0.0 (0.0–0.8)	0.0 (0.0–1.0)	0.0 (0.0–1.0)	0.0 (0.0–0.8)	0.0 (0.0–0.0)	0.0 (0.0–0.0)
Starchy vegetables	1	≥1 serve	<1 serve	0.0 (0.0–0.0)	0.0 (0.0–1.0)	0.0 (0.0–1.0)	0.5 (0.0–1.0)	0.0 (0.0–1.0)	0.0 (0.0–1.0)
Whole vegetables	1	≥1 serve	<1 serve	1.0 (1.0–1.0)	1.0 (0.0–1.0)	1.0 (0.5–1.0)	1.0 (0.0–1.0)	1.0 (0.2–1.0)	1.0 (0.5–1.0)
Legumes as vegetables	1	≥0.5 serve	<0.5 serve	0.0 (0.0–0.0)	0.0 (0.0–1.0)	0.0 (0.0–1.0)	0.0 (0.0–0.8)	0.5 (0.0–1.0)	0.0 (0.0–0.0)
Total fruit	5	≥2 serves	<0.5 serve	1.3 (0.0–2.5)	1.3 (1.3–5.0)	3.8 (0.0–5.0)	1.9 (0.0–5.0)	2.5 (1.6–3.4)	1.3 (0.6–5.0)
Fruit variety	5	2 or more varieties per day	<2 varieties per day	0.0 (0.0–0.0)	0.0 (0.0–0.0)	0.0 (0.0–5.0)	0.0 (0.0–5.0)	0.0 (0.0–0.0)	0.0 (0.0–3.0)
Total cereals	5	≥6 serves	<1 serve	2.9 (1.1–4.7)	1.7 (0.8–2.5)	4.2 (2.5–4.2)	4.2 (2.7–5.0)	4.2 (3.6–5.0)	5.0 (3.8–5.0)
Wholegrains	5	≥3 serves	<1 serve	1.5 (0.0–4.5)	0.0 (0.0–3.0)	2.0 (0.5–4.0)	2.0 (0.3–4.8)	3.5 (1.3–5.0)	3.0 (1.5–5.0)
Meat and alternatives	10	Male: ≥3 serves Female: ≥2.5 serves	Male: <1 serve Female: <0.5 serve	7.0 (1.0–10.0)	6.0 (4.0–10.0)	6.0 (4.0–10.0)	9.0 (4.0–10.0)	10.0 (8.5–10.0)	10.0 (6.0–10.0)
Dairy and alternatives	10	≥2.5 serves	<0.5 serve	7.0 (2.5–10.0)	4.0 (0.0–8.0)	8.0 (3.0–10.0)	8.0 (6.0–10.0)	10.0 (1.0–10.0)	8.0 (7.0–10.0)
% Water of total beverages	5	≥50% of total beverage volume	<10% of total beverage volume	0.0 (0.0–3.8)	0.0 (0.0–0.0)	0.0 (0.0–0.0)	0.0 (0.0–0.0)	0.0 (0.0–0.0)	0.0 (0.0–5.0)
% Energy saturated fat	5	≤10% of total energy	>12% total energy	0.0 (0.0–3.8)	0.0 (0.0–5.0)	0.0 (0.0–5.0)	1.3 (0.0–5.0)	1.3 (0.0–2.5)	2.5 (0.0–2.5)
MUFA/PUFA	5	Male: ≥4 serves Female: ≥2 serves	Male: <1 serve Female: <0.5 serve	4.4 (2.8–5.0)	5.0 (5.0–5.0)	5.0 (3.1–5.0)	5.0 (4.1–5.0)	5.0 (4.1–5.0)	2.5 (1.9–5.0)
Sodium	10	<1610 mg	≥2300 mg	7.5 (1.3–10.0)	5.0 (0.0–10.0)	0.0 (0.0–5.0)	0.0 (0.0–5.0)	0.0 (0.0–7.5)	0.0 (0.0–5.0)
Added sugars	10	<15% of total energy	>20% of total energy	10.0 (10.0–10.0)	10.0 (10.0–10.0)	10.0 (10.0–10.0)	10.0 (10.0–10.0)	10.0 (10.0–10.0)	10.0 (10.0–10.0)
Alcohol	5	≤2 standard drinks per day	>2 standard drinks per day	5.0 (5.0–5.0)	5.0 (5.0–5.0)	5.00 (5.0–5.0)	5.0 (1.3–5.0)	5.0 (5.0–5.0)	5.0 (5.0–5.0)
Total score ^a^	100			59.6 (47.2–72.0)	56.2 (46.14–66.2)	57.5 (48.6–66.4)	55.4 (48.3–62.6)	59.9 (54.0–65.8)	61.4 (52.3–70.4)

MUFA; monounsaturated fatty acid, PUFA; polyunsaturated fatty acid. Nonparametric data expressed as median (25–75 percentile). Data expressed as median (25–75 percentile), ^a^ Parametric data expressed as mean (95% CI).

**Table 4:** Nutrient intake for endurance athletes from groups A, B, and C during peak training and taper periods.

	Group A (11 and 22 km) PEAK	Group A (11 and 22 km) TAPER	Group B (50 km) PEAK	Group B (50 km) TAPER	Group C (100 km) PEAK	Group C (100 km) TAPER
**Energy total (kJ) ^**	8477 (4747–13,497)	6371 (5300–6504) ^†,#^	10,233 (7365–11423)	12,333 (8676–14,784) ^†^	11,628 (6864–15,198)	9809 (8562–12,330) ^#^
**kJ/kg**	109 (49–168)	76 (65–118) ^†^	155 (117–193)	184 (130–224) ^†^	161 (114–207)	158 (121–197)
**% Energy carbohydrate ^**	32 (31–35)	33 (29–40)	42 (37–47)	42 (37–47)	40 (32–51)	41 (30–52)
**% Energy protein**	21 (14–28)	21 (19–23)	18 (15–20)	17 (14–20) ^§^	19 (17–22)	22 (18–27) ^§^
**% Energy fat**	43 (32–54)	42 (37–47)	34 (30–38)	36 (32–40)	37 (30–45)	34 (24–45)
**% Energy fibre**	2 (2–3)	3 (1–4)	3 (2–4)	3 (2–3)	2 (2–3)	2 (1–3)
**% Energy alcohol ^**	0 (0–3)	0 (0–0)	0 (0–5)	0 (0–6)	0 (0–0)	0 (0–0)
**Carbohydrate (g) ^**	159 (93–280)	127 (118–132) ^†,#^	228 (177–321)	309 (222–371) ^†^	238 (202–390)	238 (189–322) ^#^
**g CHO/kg**	2.2 (0.8–3.5)	1.7 (1.4–2.3) ^†^	4.0 (2.7–5.4)	4.5 (2.9–6.2) ^†^	4.0 (2.4–5.6)	3.9 (2.7–5.2)
**Protein (g) ^**	99 (66–150)	76 (49–104)	100 (84–115)	127 (92–162)	142 (80–174)	135 (111–158)
**g protein/kg**	1.3 (0.5–2.2)	1.1 (0.2–2.0)	1.6 (1.2–2.5)	1.9 (1.4–2.5)	1.9 (1.3–2.5)	2.1 (1.6–2.6)
**Total fat (g) ^**	102 (49–161)	70 (56–76) ^†^	79 (62–107)	100 (88–140) ^†^	113 (66–144)	76 (72–129)
**Saturated fat (g)**	36 (4–76)	23 (3–49)	31 (24–39)	39 (30–47)	40 (28–51)	37 (18–55)
**Polyunsaturated fat (g) ^**	15 (7–24)	11 (7–17)	14 (12–19)	20 (12–30)	18 (12–27)	10 (10–19)
**Monounsaturated fat (g)**	47 (21–64)	24 (21–34)	30 (26–41)	43 (34–53)	43 (25–53)	32 (28–49)
**Trans fat (g)**	1.1 (0.0–2.1)	1.1 (1.0–3.2)	1.3 (0.9–1.7)	1.8 (1.2–2.3)	1.9 (1.2–2.5)	2.0 (0.7–3.1)
**Cholesterol (mg)**	513 (224–803) ^†^	544 (331–758)	229 (148–311) ^†,§^	325 (168–482)	478 (294–662) ^§^	398 (249–547)
**Added sugars (g) ^**	12.6 (4.5–30.3)	14.0 (11.5–25.0)	33.1 (17.1–55.1)	57.8 (24.7–121.7)	36.9 (12.9–58.1)	13.0 (9.9–42.0)
**Alcohol (g) ^**	0 (0–7)	0 (0–0)	0 (0–19)	0 (0–25)	0 (0–0)	0 (0–0)
**Dietary fibre (g) ^**	22.6 (16.9–34.5)	16.5 (16.4–23.9)	29.6 (24.2–43.8)	35.0 (24.4–53.0)	28.4 (20.5–38.5)	26.0 (18.3–33.8)
**Thiamin (mg) ^**	1.4 (0.8–1.9)	0.9 (0.5–1.6)	1.4 (1.2–6.5)	1.8 (1.2–2.3)	2.1 (1.0–4.9)	1.5 (1.0–1.6)
**Riboflavin (mg)**	1.6 (1.0–2.4)	1.2 (1.0–1.7)	1.7 (1.4–2.9)	2.3 (2.0–2.8)	2.4 (2.0–4.2)	2.2 (1.8–2.8)
**Niacin (mg)**	26.2 (3.0–49.3)	18.0 (–3.5–39.5)	32.3 (20.3–44.2)	39.3 (27.9–50.7)	32.9 (18.3–47.5)	29.9 (21.9–37.8)
**Vitamin C (mg) ^**	60.6 (31.0–130.4)	55.9 (42.1–96.8)	86.8 (49.4–272.4)	149.6 (63.9–215.9)	63.3 (33.5–91.8)	76.5 (19.6–287.3)
**Vitamin E (mg) ^**	16.8 (6.9–31.0)	7.0 (6.4–20.1)	10.8 (9.1–17.4)	14.5 (9.2–30.0)	19.2 (9.7–37.1)	10.46 (8.86–22.8)
**Vitamin B12 (μg) ^**	3.6 (3.2–3.9)	3.3 (3.2–4.6)	4.7 (3.6–12.7)	6.7 (3.7–12.6)	8.6 (5.5–12.2)	6.2 (6.1–7.6)
**Folate (μg) ^**	513 (321–432)	391 (77–704)	563 (432–675)	618 (463–773)	598 (456–849)	571 (361–780)
**Beta Carotene (μg) ^**	3499 (1521–11412)	4361 (2889–8809)	7122 (1246–15967)	4486 (1541–14658)	1231 (519.4–3493)	2982 (1107–4012)
**Sodium (mg)**	2626 (−723.1–5976)	2168 (−841.1–5178)	2841 (1969–3713)	2915 (2314–3515)	3029 (1829–4229)	2468 (2150–2786)
**Potassium (mg) ^**	3685 (2288–4973)	2710 (993.5–4427)	3972 (3069–4604)	5213 (3604–6822)	4270 (3089–5019)	4078 (3224–4931)
**Magnesium (mg) ^**	340 (221–668)	216 (166–285) ^†,#^	453 (348–540)	528 (444–638) ^†^	461 (293–730)	462 (347–542) ^#^
**Calcium (mg)**	843 (241–1445)	691 (250–1132)	1211 (813–1608)	1266 (975–1558)	1327 (806–1847)	952 (709–1196)
**Iron (mg) ^**	10.2 (7.2–16.6)	7.9 (7.8–9.7) ^†,#^	12.1 (10.0–13.0)	16.2 (11.2–23.1) ^†^	16.0 (10.3–16.6)	15.6 (11.4–18.1) ^#^
**Zinc (mg)**	10.2 (1.5–20.0)	6.2 (3.5–8.8)	13.1 (8.9–17.3)	16.8 (11.5–22.0)	15.5 (10.3–20.8)	19.4 (13.1–25.8)
**Iodine (μg)**	192 (38–345)	150 (145–173)	178 (135–221)	147 (108–214)	243 (157–329)	201 (150–241)
**Selenium (μg) ^**	95 (74–122)	79 (8–150)	68 (55–111)	114 (78–151)	111 (63–139)	79 (48–111)
**Linoleic (g) ^**	12.8 (5.4–21.0)	8.5 (5.4–11.4)	12.2 (9.6–17.1)	16.7 (10.3–25.7)	14.1 (10.1–23.2)	9.0 (7.8–14.9)
**Alpha-linolenic (ALA) (g) ^**	2.0 (0.8–2.7)	1.9 (1.0–2.9)	1.5 (1.1–1.9)	2.2 (1.4–3.7)	1.6 (1.0–2.4)	1.0 (0.8–2.2)
**Eicosapentaenoic (EPA) (mg) ^**	26.1 (6.9–62.9)	8.1 (1.3–49)	29.8 (14.8–292.9)	9.1 (0.3–13.6)	125.3 (21.4–391.6)	5.3 (4.8–45.1)
**Docosapentaenoic (DPA) (mg) ^**	66.7 (29.1–112.1)	3.6 (3.5–20.98)	46.8 (26.0–104.9)	6.8 (2.9–19.8)	94.1 (51.3–262.9)	12.1 (6.7–30.4)
**Docosahexaenoic (DHA) (mg) ^**	83.6 (39.4–137.7)	26.3 (4.7–108.7)	46.1 (14.8–405.2)	6.5 (0.1–34.2)	138.5 (26.7–577.5)	2.6 (1.4–60.5)

Data expressed as mean (95%CI); ^^^ Nonparametric data expressed as median (25–75 percentile); Within time points of PEAK or TAPER the following symbols indicate, ^†^ significant difference between groups A & B; *p* < 0.05, ^§^ significant difference between groups B & C; *p* < 0.05, ^#^ significant difference between groups A & C; *p* < 0.05.

## Data Availability

The raw data supporting the conclusions of this article will be made available by the authors on request.

## Abbreviations

The following abbreviations are used in this manuscript:

CHO	carbohydrates
MUFA	monounsaturated fatty acid
PUFA	polyunsaturated fatty acid
AUSNUT	AUStralian Food and NUTrient Database
HEIFA	Healthy Eating Index for Australian Adults
DHA	docosahexaenoic acid
EPA	eicosapentaenoic acid
T2D	type 2 diabetes
CVD	cardiovascular disease
ISSN	International Society of Sports Nutrition
IOC	International Olympic Committee
DQI	Diet Quality Index
